# Lateralized Difference in Tympanic Membrane Temperature: Emotion and Hemispheric Activity

**DOI:** 10.3389/fpsyg.2013.00104

**Published:** 2013-03-04

**Authors:** Ruth E. Propper, Tad T. Brunyé

**Affiliations:** ^1^Cerebral Lateralization Laboratory, Psychology Department, Montclair State UniversityMontclair, NJ, USA; ^2^Department of Psychology, Tufts UniversityMedford, MA, USA; ^3^Cognitive Science Team, U.S. Army Natick Soldier Research, Development and Engineering CenterNatick, MA, USA

**Keywords:** tympanic membrane temperature, motivational orientation, cortical asymmetry, lateralization, mood, emotion, hemispheric asymmetry

## Abstract

We review literature examining relationships between tympanic membrane temperature (TMT), affective/motivational orientation, and hemispheric activity. Lateralized differences in TMT might enable real-time monitoring of hemispheric activity in real-world conditions, and could serve as a corroborating marker of mental illnesses associated with specific affective dysregulation. We support the proposal that TMT holds potential for broadly indexing lateralized brain physiology during tasks demanding the processing and representation of emotional and/or motivational states, and for predicting trait-related affective/motivational orientations. The precise nature of the relationship between TMT and brain physiology, however, remains elusive. Indeed the limited extant research has sampled different participant populations and employed largely different procedures and measures, making for seemingly discrepant findings and implications. We propose, however, that many of these discrepancies can be resolved by considering how emotional states map onto motivational systems, and further examining how validated methods for inducing lateralized brain activity might affect TMT.

## Introduction

The development of techniques allowing examination of brain activity in “real-time” has enabled great strides to be made in the field of behavioral neuroscience generally, and in determining the cortical substrates of emotion and motivational orientation specifically. Electroencephalography and functional imaging have indicated broad differences in hemispheric lateralization of affect/motivation, with the right hemisphere being specialized for negative affect and withdrawal motivation, and the left hemisphere being specialized for positive affect and approach motivation (e.g., Davidson, 2002, 2004; Davidson et al., 1990; Tomarken et al., 1992).

Unfortunately, EEG and functional imaging techniques are expensive, time consuming, limited, or impossible for use in real-world situations, and require specialized knowledge. In order to remove some of these limitations, other methods to assess these relationships have been investigated. One such method is lateralized differences in tympanic membrane temperature (TMT).

Tympanic membrane temperature may reflect hemispheric activity in frontal and temporal lobes (e.g., Schiffer et al., 1999). Increased hemispheric activity is associated with increased propensity to experience that hemisphere’s affective/motivational state. For example, individuals with increased right hemisphere activity are more likely to experience negative affect and withdrawal motivation (e.g., Henriques and Davidson, 1991), while those with increased left hemisphere activity are more likely to experience positive affect and approach motivation (e.g., Davidson, 2004). TMT, by reflecting hemispheric activity, may therefore be predictive of emotional and motivational state.

Unfortunately, however, the relationship between TMT and hemispheric activity is by no means straightforward. Although discussion of the physiological mechanisms whereby TMT changes as a function of hemispheric activity is beyond the scope of this mini-review, it should be noted that there are several contending, and contradictory, hypotheses. For simplicity, physiological mechanisms can be grouped into two broad schools of thought, leading to opposite predictions of the relationship between TMT and emotion/motivation; (i) increased TMT on one versus the other side is associated with ipsilaterally *increased* hemispheric activity (e.g., Boyce et al., 2002; Gunnar and Donzella, 2004) and (ii) increased TMT on one versus the other side is associated with ipsilaterally *decreased* hemispheric activity (Boyce et al., 1996; Helton, 2010; Helton et al., 2009a). Thus, based on proposed physiological mechanisms alone it is not clear *a priori*, in any given circumstance, if, for example, *increased* left TMT is associated with positive emotion [via (i) above] or with negative emotion [via (ii) above].

Examination of the TMT-affect/motivation literature could help elucidate TMT-hemispheric activity relationships, and shed light on the physiological mechanisms that underlie them. Additionally, should a clear predictive relationship between TMT and affect/motivation exist, such a finding would be eminently useful for both research and clinical purposes. From a research perspective, lateralized differences in TMT might enable real-time monitoring of hemispheric activity in real-world conditions. From a clinical perspective, lateralized differences in TMT might serve as a corroborating marker of mental illnesses associated with specific affective dysregulation.

Thus, the purpose of this mini-review is to (i) determine if lateralized differences in TMT are systematically related to emotion/motivation and (ii) if so, by inference, is increased TMT associated with ipsilaterally *increased* or with ipsilaterally *decreased* hemispheric activity.

## The Literature

A review of the literature revealed a total of 12 articles (including one in press from the authors’ laboratories) meeting the following criteria: (i) use of TMT as a dependent measure, (ii) affect or approach/withdrawal behavior as either dependent or independent variables; (iii) analyses allowing determination of affect or approach/withdrawal-TMT relationships; and (iv) humans as participants. Articles were found via a search of PubMed, using various combinations of the key words (and their main root word): “tympanic,” “temperature,” “emotion,” “mood,” “lateralization,” “asymmetry,” and “motivation.” Articles were also found via citations listed in articles found using the above methodology.

Some articles, although including TMT and affect/motivation, were eliminated because they failed to meet one of the above criteria (e.g., Schiffer et al., 1999; Parr and Hopkins, 2000; Cherbuin and Brinkman, 2004).

A summary of the results is presented in Table 1.

**Table 1:** **Tympanic membrane temperature and affect/motivational orientation articles**.

Article	Participant age	Task or report	Main findings	Summary
Boyce et al. (2002)	4.5–8 year olds	Parental report of affect/behavior	Increased L-R TMT associated with positive emotions and socially “competent” behaviors; decreased L-R TMT associated with negative emotions and “problem” behaviors	Warmer L TMT associated with positive/approach emotions; warmer R TMT associated with negative emotions
Boyce et al. (1996)	8 year olds	Parental report of affect/behavior	Increased L-R TMT associated with increased aggression, decreased ego resilience, depression, externalizing, and internalizing behavior problems, schizoid behaviors, social withdrawal, and somatization	Warmer L TMT associated with negative/withdrawal emotions
Gunnar and Donzella (2004)	3–5 year olds	Parental report of affect/behavior	Increased L-R TMT associated with increased smiling and laugher, decreased L-R TMT associated with increased sadness	Warmer L TMT associated with positive/approach emotions; warmer R TMT associated with negative emotions
Helton (2010)	19.97 Mean age (undergraduates)	Impulsivity assessed via performance on Go-No-Go tasks	Warmer L TMT associated with increased errors of commission and faster reaction time	Warmer L TMT associated with increased impulsivity/approach motivation; Increased R TMT associated with more “cautious” behavior
Helton and Carter (2011)	20.3 Mean age (undergraduates)	TMT assessed at baseline by male or female experimenter	Lower R TMT when measured by female investigator; Similar R and L TMT when measured by male investigator	Placed in framework of “threat assessment”; interpretation not clear
Helton et al. (2009a)	Range of 18–33 years	Attention assessed via performance on local and global sustained attention tasks	Increased R TMT associated with sustained attention to global stimuli	Warmer R TMT associated with global attention; interpreted as cognitive fatigue-related decreased R hemisphere activity
Helton et al. (2009b)	20 Mean age (undergraduates)	No affect report following possible mood induction, attention assessed via performance on the sustained attention to response task	Greater increase in R TMT compared to L TMT from pre to post-task	Warmer R TMT associated with increased attentiveness
Helton and Maginnity (2012)	21 Mean age (undergraduates)	Self-report of attention/inattention	Increased R-L TMT associated with decreased inattention	Warmer R TMT associated with increased attentiveness
Jones et al. (2011)	7–10 year olds	No affect report following stress induction	Low fetal-maternal “health” at birth associated with increased L-R TMT following stress	Warmer L TMT associated with negative emotions
Propper et al. (2010)	19.66 Mean age (undergraduates)	Self-report of affect at baseline	No association between R-L TMT and any emotion; Ar-ITMT associated with increased anger/hostility	Greater absolute *difference* between L and R TMT associated with anger/hostility
Propper et al. (in press)	Undergraduates (age not reported)	Self-report of affect following mood induction	Baseline: increased R TMT associated with increased positive affect; post-manipulation: increased R TMT associated with increased negative affect; increased L TMT associated with positive affect	Greater absolute *difference* between L and R TMT associated with anger/hostility; other results not clear
Propper et al. (2011)	Range of 19–21 years per group (undergraduates)	Self-report of affect following sustained unilateral visual stimulation	Increased Ar-ITMT associated with increased anger/hostility, increased R-L TMT associated with increased anger	Greater absolute *difference* between L and R TMT associated with anger/hostility; warmer R TMT associated with negative emotions

## Results

### General

Twelve articles were produced by five laboratories. One laboratory (Helton and colleagues) produced 42% (5), while another (Propper and colleagues) produced 25% (3). An additional laboratory produced 17% (2; Boyce and colleagues). The remaining two manuscripts came from two different laboratories (Gunnar and Donzella, 2004; Jones et al., 2011). Of the 75% (9) published after or during 2010, all but one were from the laboratory of either Helton or Propper. These results suggest that investigation of TMT-emotion/motivation is in its infancy, and that while any consistent findings may be promising, they will need to be replicated and extended by other laboratories.

Thirty-three percent (4) examined children 3–9 years of age; the other 67% (8) investigated TMT in college students. It is not clear if results of young children are generalizable to adults; brain organization, including lateralization of cerebral functions, in addition to structural organization, is subject to developmental changes (e.g., Groen et al., 2012). Because so few TMT studies exist, future research will need to address whether the same processes underlying any TMT-affect/motivational relationships are the same in children and adults.

### Studies in children

Three out of four (75%) studies examining children relied on parental reports of affect/motivational orientation. The fourth (Jones et al., 2011), although investigating TMT in response to stress, did not directly assess affect or motivation, but is included below because it is relevant to the issues discussed here.

The earliest study (Boyce et al., 1996) examined parental reports of behavior and emotional orientation in 8 year old children. Boyce reported that warmer left, relative to right TMT was associated with “decreased ego resilience.” Further, examination of their results indicates that increased aggression, externalizing and internalizing behavioral problems, somatization, schizoid behaviors, depression, and social withdrawal were also associated with increased left TMT. Boyce et al. suggested that increased lateralized TMT was related to ipsilaterally *decreased* hemispheric activity.

Boyce et al. (2002), in an extension and replication, examined TMT-affect/motivation relationships in four cohorts of children ages 4.5–8 years old. In contrast with Boyce et al. (1996), here it was reported that warmer left, relative to right, TMT was associated with positive affect/approach motivation, and warmer right, relative to left TMT was associated with negative affect/withdrawal motivation. Thus, these results suggest the converse of those described above, with warmer left TMT reflecting increased left hemisphere activity, and increased lateralized TMT related to ipsilaterally *increased* hemispheric activity.

It is not clear why there exists a discrepancy between the two studies; both examined children approximately the same age, and both relied on parental reports of affect/motivational orientation. However, it is notable that Boyce et al. (1996) characterized “aggression” and “problem behaviors” as negative affect/withdrawal motivation. As indicated in Table 2, recent work indicates that “aggression” may be considered an approach motivational state. Additionally, by definition behavioral disinhibition and impulsivity such as that reflected in “externalizing and internalizing behavioral problems,” somatization, and schizoid behaviors may be considered left hemisphere, approach behaviors. Though it is not clear why social withdrawal and depression would be associated with increased left TMT, most results reported by Boyce et al. (1996), when considered in this manner, are in agreement with Boyce et al., 2002, such that increased left TMT reflects increased left hemisphere activity.

**Table 2:** **Affective valence × motivational state**.

		Motivational state	
		Approach	Avoidance
Affective valence	Positive	Enthusiasm Amusement Desire/pleasure	–
	Negative	Anger Frustration Aggression	Anxiety Fear Disgust

Gunnar and Donzella (2004), examining parental reports of affect and motivational orientation in 3–5 year olds, reported findings consistent with Boyce et al. (2002); warmer left, relative to right, TMT was associated with positive affect, and warmer right, relative to left TMT was associated with negative affect. Specifically, higher scores on a smiling and laughing scale were associated with warmer left TMT, while higher scores on the sadness scale were associated with warmer right TMT. Interestingly, score on the fear, anger, and shyness scales were not correlated with differential left or right TMT.

Jones et al. (2011) examined TMT in 8 and 9 year old children following a psychosocial stressor. Both left and right TMT decreased following the stressor, and there were no differences between left and right TMT. Birth weight adjusted for placental weight in the children (from previously collected longitudinal data) did correlate with left TMT after stress; children who were smaller babies with bigger placentas had higher left TMT following stress, while larger babies with smaller placentas had warmer right TMT following stress. The authors interpreted their findings as indicating *increased* right hemisphere activity following stress in children with small sizes and larger placentas.

That is, findings were put into a framework wherein increased lateralized TMT is associated with *decreased* ipsilateral hemispheric activity. However, it is interesting to note that (i) no measures of stress or mood were examined and (ii) both left and right TMT decreased equally following stress. Additionally, individual differences in birth weight-placental size may be associated with individual differences in any number of other areas, including structural and functional cerebral lateralization, or in overall subjective response to “stress.” If so, then the results indicate a relationship such that increased lateralized TMT is associated with ipsilaterally *increased* hemispheric activity.

The TMT-affect/motivation literature in children offers some limited evidence that (i) warmer left TMT is associated with positive affect/approach motivational states and that warmer right TMT is associated with negative affect/withdrawal motivational states; and (ii) a relationship between lateralized TMT and affect/motivation such that increased lateralized TMT is associated with increased ipsilateral hemispheric activity.

### Studies in adults

The 67% (8) studies examining TMT-affect/motivation in adults come from two laboratories, Helton and colleagues and Propper and colleagues. All were published in 2010 or later, and all examined college students.

Helton et al. (2009a), Helton and Maginnity (2012), and Helton et al. (2009b) examined adult college students, using self-reported (Helton and Maginnity, 2012) and performance measures (Helton et al., 2009a; Helton et al., 2009b) of attention. One interpretation of focused or sustained attention is that it represents a state of approach motivation (Gable and Harmon-Jones, 2008). In all three studies, warmer right TMT was associated with increased attention. Helton et al. (2009a), investigated sustained attention in a paradigm examining local and global processing. They reported increased right TMT to be associated with increased sustained attention; they interpreted their findings as indicative of right hemisphere fatigue, and of decreased right hemisphere activity. Helton et al. (2009b) reported increased right TMT following exposure to negative pictures, and additionally following performance of a sustained attention to response task, considered as a test of sustained attention. Again, results were interpreted as indicating warmer TMT being associated with *decreased* ipsilateral hemispheric activity. Finally, Helton and Maginnity (2012) reported that decreased self-reported symptoms of inattention in college students were associated with increased right TMT.

Although Helton et al. (2009a) and Helton et al. (2009b) suggest that increased right TMT is associated with decreased right hemisphere activity, alternate explanations exist. First, clear differences in attentional capacities *per se* are known to exist between the hemispheres. Specifically, the right hemisphere is known to have a much larger role in attending generally, relative to the left hemisphere (Ramachandran and Blakeslee, 1999). Additionally, the right hemisphere is known to be particularly involved in vigilance (Warm et al., 2009). Thus, when considered from a perspective other than emotion/motivation, the above results are consistent with the notion that increased TMT on one versus the other side reflects ipsilaterally *increased* hemispheric activity. Similarly, the increased right TMT following exposure to negative pictorial stimuli supports this interpretation, given the right hemisphere’s known role in negative affect/withdrawal motivation, and the results reported in studies of children’s TMT.

Helton (2010) examined college students, using behavioral measures of impulsivity/approach motivation in an experimental design. In a Go-No-Go task, increased errors of commission, and faster reaction times, were associated with increased left TMT. Findings were interpreted such that increased impulsivity and approach motivation were associated with increased left TMT, and that increased lateralized TMT was indicative of *increased* ipsilateral hemispheric activity.

Helton and Carter (2011) reported effects of experimenter gender on lateralized TMT, finding warmer left, relative to right TMT when participants were tested by a female investigator. They interpreted their findings as resulting from “threat appraisal,” with decreased threat detected with female researchers. Although only minimally discussed, it was suggested that warmer TMT is associated with ipsilaterally *decreased* hemispheric activity; in this case then, decreased threat is associated with increased right hemisphere activity. However, it is equally plausible that the presence of a female investigator increased approach motivation (relative to a male investigator), and that therefore increased left TMT is associated with ipsilaterally *increased* hemispheric activity.

Work from the authors’ laboratories (Propper et al., 2010, 2011, in press) examined self-reported affect at baseline and following experimental manipulations. Propper et al. (2010) examining baseline TMT-affect relationships reported that greater difference between the left and right TMT was associated with increased anger. No other TMT-affect relationships attained significance.

Propper et al. (2011), manipulated hemispheric activity via sustained unilateral gaze to examine effects on TMT. Increased left TMT was associated with increased anger collapsing across all conditions, as was the absolute difference between the left and right TMT. Though interpreted as indicating an association between negative affect (anger) and increased right hemisphere activity (that is, ipsilaterally *decreased* hemispheric activity), given research indicating that anger is an approach motivational state (Harmon-Jones and Allen, 1998), the findings are at least equally likely to suggest that increased lateralized TMT is associated with increased ipsilateral hemispheric activity. Propper et al. (in press), examined TMT following mood induction. Findings again indicated that increased difference between the left and right TMT, regardless of the direction of that difference, was associated with increased anger. Additionally, tentative evidence was presented suggesting pre versus post-task differences in the relationship between TMT and affect/motivational state, such that pre-task lateralized TMT is associated with ipsilaterally *decreased* hemispheric activity, but post-task with ipsilaterally *increased* hemispheric activity. However, the pre-task findings occurred only in men, with few participants, and in only one mood (happiness/sadness) condition. In contrast, post-task associations occurred for both calm/anxious mood and for happiness/sadness, such that lateralized TMT was associated with ipsilaterally increased hemispheric activity.

## Summary

The present review supports the proposal that TMT holds potential for broadly indexing lateralized brain physiology during tasks demanding the processing and representation of emotional and/or motivational states, and for predicting trait-related affective/motivational orientations. The precise nature of the relationship between TMT and brain physiology, however, remains elusive. Indeed the limited extant research has sampled different participant populations and employed largely different procedures and measures, making for seemingly discrepant findings and implications. For example, methodologically, some of the studies reviewed here examined TMT as an indicator of stable, between-subjects individual differences in affective/motivational orientation (e.g.: Boyce et al., 1996; Boyce et al., 2002; Propper et al., 2010; Helton and Maginnity, 2012), while others considered TMT as indicating within-subjects variation in affective/motivational state (e.g., Helton et al., 2009a,b; Propper et al., 2011, in press). Still others examined TMT as reflecting trait differences *in response* to state manipulations involving stress (e.g., Jones et al., 2011). Interestingly, it has been proposed that within- versus between-subjects examinations may influence TMT-hemispheric relationships, with increased TMT being associated with (i) decreased ipsilateral hemispheric activity in within-subjects designs and (ii) increased ipsilateral hemispheric activity in between-subjects designs. (e.g., Cherbuin and Brinkman, 2007; Helton, 2010; Propper et al., in press). However, the literature reviewed here reveals no clear pattern for differences in lateralized TMT as a function of consideration of state versus trait variables, and we leave it to others to investigate this suggestion further empirically.

We do propose, however, that many of the discrepancies in the TMT literature can be resolved by considering how emotional states map onto motivational systems (i.e., Table 2), and further examining how validated methods for inducing lateralized brain activity might affect TMT.

Traditional theories attempting to define emotional experience considered emotions as modular entities (e.g., Izard, 1991; Ekman, 1992), or sought to describe their underlying dimensions of valence and arousal (Lang et al., 1992; Russell, 2003). More recent work has demonstrated utility in defining not only the modularity of basic emotions or their underlying valence and arousal, but also considering the nature of events that elicit and reinforce emotions. The events associated with particular emotions carry underlying states that motivate certain types of behavior (Frijda, 1986; Davidson, 1998). In general, these motivational states are associated with approach or avoidance motives, both considered core elements in the organization of human behavior (Carver and Harmon-Jones, 2009). Critically, approach and avoidance motivations are reliably associated with distinct neural substrates thought to reside in the left and right brain hemispheres, respectively (Davidson, 1998; Pizzagalli et al., 2005). Thus, while affective states such as anger and fear are decidedly negative in valence and high arousal, they are associated with opposing motivational systems; anger promotes approach-related motives whereas fear promotes avoidance. With these distinctions in mind, we have suggested that many seemingly discrepant results can be at least partially resolved by considering how individual emotions map onto underlying motivational states.

More difficult to reconcile, however, are mixed reports detailing the directionality of any relationships between TMT and hemispheric activity. Several studies suggest that increased TMT is associated with decreased ipsilateral hemispheric activity (e.g., Helton et al., 2009a,b; Helton and Maginnity, 2012); others suggest the opposite (Helton and Carter, 2011). Still others suggest that hemispheric differences in TMT, regardless of direction, might reliably indicate the presence of an approach-oriented emotion such as anger (Propper et al., 2010, in press). Future research might attempt to replicate this latter effect with emotional states characterized by opposing motivations, such as fear. Individual differences in lateralization of emotion may have contributed to inconsistent relationships; research might control for these individual differences by ensuring that all participants are strongly right-handed.

We propose at least three types of continuing research that might prove successful in further detailing the relationship between TMT and emotional and motivational states. First, we propose that if TMT can be used to reliably index hemispheric activity, then actively promoting neural activity in specific hemispheres, such as via sustained unilateral gaze or trans-cranial direct current stimulation (tDCS), should elicit reliable differences in TMT. Some recent work in our laboratories suggests that this might be the case (Propper et al., 2011). Low-current brain stimulation techniques, such as tDCS might also prove useful in temporarily increasing neural activity in one or both brain hemispheres. Second, we propose functional neuroimaging techniques such as EEG should be used to assess potential relationships between TMT, affect, and process-specific frequency bands. As reviewed above, we are aware of only one such study (Schiffer et al., 1999). Third, we propose value to continuing research examining motivational states associated with particular emotions; for instance, studies examining TMT responses to viewing stimuli reliably associated with appetitive (approach-related) versus disgusting (avoidance-related) motives.

Finally, we’d like to point out that not only may emotional/motivational states be lateralized, but so too are many cognitive processes (e.g., Hellige, 2001), offering the possibility that TMT may also reflect cognition. In fact, research suggests that TMT may reflect lateralized cognitive performance in some domains. For example, performance on a visuo-spatial task demonstrated decreased TMT, while performance on a verbal task resulted in decreased left TMT (Cherbuin and Brinkman, 2004). Thus, TMT, via being indicative of hemispheric activity, may also have potential in investigations of lateralized cognitive activity.

## Conclusion

Sampling brain activity in “real-time” using portable and non-invasive technologies holds promise for understanding neurophysiologic correlates of real-world experiences. Though very few studies have examined whether TMT might hold value toward this goal, the extant data suggest that TMT is indeed sensitive to manipulations of emotional and/or motivational states. Precisely defining these relationships will be a critical goal for continuing research in this exciting area.

## Conflict of Interest Statement

The authors declare that the research was conducted in the absence of any commercial or financial relationships that could be construed as a potential conflict of interest.

## References

[B1] Boyce W. T. Essex M. J. Alkon A. Smider N. A. Pickrell T. Kagan J. (2002). Temperment, tympanum, and temperature: four provisional studies of the biobehavioral correlates of tympanic membrane temperature asymmetries. Child Dev. 73, 718–733 10.1111/1467-8624.00434 12038547

[B2] Boyce W. T. Higley D. Jemerin J. J. Champoux M. Suomi S. J. (1996). Tympanic temperature asymmetry and stress behavior in Rhesus Macaques and children. Arch. Pediatr. Adolesc. Med. 150, 518–523 10.1001/archpedi.1996.02170300072014 8620235

[B3] Carver C. S. Harmon-Jones E. (2009). Anger is an approach-related affect: evidence and implications. Psychol. Bull. 135, 183–204 10.1037/a0015026 19254075

[B4] Cherbuin N. Brinkman C. (2004). Cognition is cool: can hemispheric activation be assessed by tympanic membrane thermometry? Brain Cogn. 54, 228–231 10.1016/j.bandc.2004.02.014 15050780

[B5] Cherbuin N. Brinkman C. (2007). Sensitivity of functional tympanic membrane thermomentry (fTMT) as an index of hemispheric activation in cognition. Laterality 12, 239–261 17454574 10.1080/13576500701218345

[B6] Davidson R. J. (1998). Affective style and affective disorders: perspectives from affective neuroscience. Cogn. Emot. 12, 307–320 10.1080/026999398379600

[B7] Davidson R. J. (2002). Anxiety and affective style: role of prefrontal cortex and amygdala. Biol. Psychiatry 51, 68–80 10.1016/S0006-3223(01)01328-2 11801232

[B8] Davidson R. J. (2004). Well-being and affective style: neural substrates and biobehavioral correlates. Philos. Trans. R. Soc. Lond. B Biol. Sci. 359, 1395–1411 10.1098/rstb.2004.1510 15347531 PMC1693421

[B9] Davidson R. J. Ekman P. Saron C. D. Senulis J. A. Friesen W. V. (1990). Approach-withdrawal and cerebral asymmetry: emotional expression and brain physiology I. J. Pers. Soc. Psychol. 58, 330–341 10.1037/0022-3514.58.2.330 2319445

[B10] Ekman P. (1992). An argument for basic emotions. Cogn. Emot. 6, 169–200 10.1080/02699939208411068

[B11] Frijda N. O. (1986). The Emotions. Cambridge: Cambridge University Press

[B12] Gable P. A. Harmon-Jones E. (2008). Approach-motivated positive affect reduces breadth of attention. Psychol. Sci. 19, 476–482 10.1111/j.1467-9280.2008.02195.x 18466409

[B13] Groen M. A. Whitehouse A. J. O. Badcock N. A. Bishop D. V. M. (2012). Does cerebral lateralization develop? A study using functional transcranial Doppler ultrasound assessing lateralization for language production and visuospatial memory. Brain Behav. 2, 256–269 10.1002/brb3.56 22741100 PMC3381631

[B14] Gunnar M. R. Donzella B. (2004). Tympanic membrane temperature and emotional dispositions in preschool-aged children: a methodological study. Child Dev. 75, 497–504 10.1111/j.1467-8624.2004.00689.x 15056203

[B15] Harmon-Jones E. Allen J. J. B. (1998). Anger and frontal brain activity: EEG asymmetry consistent with approach motivation despite negative affective valence. J. Pers. Soc. Psychol. 74, 1310–1316 10.1037/0022-3514.74.5.1310 9599445

[B16] Hellige J. B. (2001). Hemispheric Asymmetry: What’s Right and What’s Left. Cambridge, MA: Harvard University Press

[B17] Helton W. S. (2010). The relationship between lateral differences in tympanic membrane temperature and behavioral impulsivity. Brain Cogn. 74, 75–78 10.1016/j.bandc.2010.06.008 20656394

[B18] Helton W. S. Carter J. R. (2011). The effect of investigator gender on lateral tympanic membrane temperature. Laterality 16, 156–163 20169500 10.1080/13576500903468896

[B19] Helton W. S. Harynen L. Schaeffer D. (2009a). Sustained attention to local and global target features is different: performance and tympanic membrane temperature. Brain Cogn. 71, 9–13 10.1016/j.bandc.2009.03.001 19329239

[B20] Helton W. S. Kern R. P. Walker D. R. (2009b). Tympanic membrane temperature, exposure to emotional stimuli and the sustained attention to response task. J. Clin. Exp. Neuropsychol. 31, 611–616 10.1080/13803390802375565 18923963

[B21] Helton W. S. Maginnity M. (2012). Increased attentiveness is associated with hemispheric asymmetry measured with lateral tympanic membrane temperature in humans and dogs. Exp. Brain Res. 219, 321–326 10.1007/s00221-012-3065-0 22488130

[B22] Henriques J. B. Davidson R. J. (1991). Left frontal hypoactivation in depression. J. Abnorm. Psychol. 100, 535–545 10.1037/0021-843X.100.4.535 1757667

[B23] Izard C. E. (1991). The Psychology of Emotions. New York: Plenum Press

[B24] Jones A. Osmond C. Godfrey K. M. Phillips D. I. (2011). Evidence for developmental programming of cerebral laterality in humans. PLoS ONE 6:e17071 10.1371/journal.pone.0017071 21359174 PMC3040213

[B25] Lang P. J. Bradley M. M. Cuthbert B. N. (1992). A motivational analysis of emotion: reflex-cortex connections. Psychol. Sci. 3, 44–49 10.1111/j.1467-9280.1992.tb00255.x

[B26] Parr L. A. Hopkins W. D. (2000). Brain temperature asymmetries and emotional perception in chimpanzees, Pan troglodytes. Physiol. Behav. 71, 363–371 10.1016/S0031-9384(00)00349-8 11150569

[B27] Pizzagalli D. A. Sherwood R. J. Henriques J. B. Davidson R. J. (2005). Frontal brain asymmetry and reward responsiveness: a source-localization study. Psychol. Sci. 10, 805–813 10.1111/j.1467-9280.2005.01618.x 16181444

[B28] Propper R. E. Brunye T. T. Christman S. D. Bologna J. (2010). Negative emotional valence is associated with non-right-handedness and increased imbalance of hemispheric activation as measured by tympanic membrane temperature. J. Nerv. Ment. Dis. 198, 691–694 10.1097/NMD.0b013e3181ef1f35 20823734

[B29] Propper R. E. Januszewski A. Brunyé T. T. Christman D. (in press). Tympanic membrane temperature, hemispheric activity, and affect: evidence for a modest relationship. J. Clin. Exp. Neuropsychol. 10.1176/appi.neuropsych.12020027 23695535

[B30] Propper R. E. Januszewski A. Christman S. D. Brunye T. T. (2011). Increased anger is associated with increased hemispheric asymmetry. J. Nerv. Ment. Dis. 199, 716–720 10.1097/NMD.0b013e318229d95a 21878789

[B31] Ramachandran V. S. Blakeslee S. (1999). Phantoms in the Brain: Probing the Mysteries of the Human Mind. New York: William Morrow

[B32] Russell J. A. (2003). Core affect and the psychological construction of emotion. Psychol. Rev. 110, 145–172 10.1037/0033-295X.110.1.145 12529060

[B33] Schiffer F. Anderson C. M. Teicher M. H. (1999). Electroencephalogram, bilateral ear temperature, and affect changes induced by lateral visual field stimulation. Compr. Psychiatry 40, 221–225 10.1016/S0010-440X(99)90007-X 10360618

[B34] Tomarken A. J. Davidson R. J. Wheeler R. E. Doss R. C. (1992). Individual difference in anterior brain asymmetry and fundamental dimensions of emotion. J. Pers. Soc. Psychol. 62, 676–687 10.1037/0022-3514.62.4.676 1583591

[B35] Warm J. S. Matthews G. Parasuraman R. (2009). Cerebral hemodynamics and vigilance. Mil. Psychol. 21(Suppl. 1), S75–S100 10.1080/08995600802554706

